# Study of the Effect of ZnO Functionalization on the Performance of a Fully Formulated Engine Oil

**DOI:** 10.3390/nano13182540

**Published:** 2023-09-11

**Authors:** Marta Hernaiz, Iker Elexpe, Estíbaliz Aranzabe, Beatriz Fernández, Xana Fernández, Silvia Fernández, Martí Cortada-García, Andrés T. Aguayo

**Affiliations:** 1 Fundación Tekniker, Inaki Goenaga 5, 20600 Eibar, Spain; iker.elexpe@tekniker.es (I.E.); beatriz.fernandez@tekniker.es (B.F.); xana.fernandez@tekniker.es (X.F.); 2 Repsol Technology Lab., Agustín de Betancourt S/N., 28935 Móstoles, Spain; silvia.fernandez@repsol.com (S.F.); marti.cortada@repsol.com (M.C.-G.); 3 Department of Chemical Engineering, University of the Basque Country UPV/EHU, P.O. Box 644, 48080 Bilbao, Spain; andrestomas.aguayo@ehu.es

**Keywords:** fuel economy, ZnO, oleic acid, surface functionalization, friction, wear, thermo-oxidative properties, viscosity, nanolubricants, engine oil

## Abstract

The automotive sector is demanding higher specifications to achieve maximum efficiency; in this sense a new generation of lubricants with higher thermo-oxidative stability and superior tribological properties is being explored. The formulation of nanolubricants based on the nature of different nanomaterials is one of the most recent approaches, with several gaps to cover, such as dispersion stability, related to the compatibility of proposed nanomaterials with conventional additives and baseoils used in lubricant formulation. This study evaluated the effect of ZnO nanomaterial dispersed in a commercial engine oil using two different approaches; the use of surfactant and nanomaterial surface functionalization to promote higher stability and lower cluster size. Experimental evidence shows a synergetic effect between the tribological protection mechanism and the antioxidant properties in the lubricant. The effect of nanoparticle cluster size, functionalization level, and nanomaterial content are presented.

## 1. Introduction

Promoting fuel economy and reducing emissions are the main objectives of the automotive industry. Increasing the efficiency of internal combustion engines (ICEs) is one of the most promising and cost-effective approaches to dramatically improve the fuel economy of the on-road vehicle fleet in the short to medium term. To this end, reducing mechanical losses through improved lubrication systems is essential [1].

Lubrication plays a key role in both performance and engine life, and formulators must design lubricants with high thermo-oxidative stability and superior tribological properties to cope with increasingly extreme operating conditions (high loads and speeds), especially for new engines [2].

In recent years, with the advancements of nanotechnology, nanomaterials (NMs) have emerged as potential tribological additives, reducing friction and increasing wear protection in sliding contacts [3,4]. Tribological enhancement through the use of NM can involve three types of mechanisms: a physical deposition mechanism that promotes a rolling sliding effect, a chemical reaction mechanism forming a tribofilm, and a self-repair mechanism by promoting surface mending effect [5].

Along with the improvement of the tribological behaviour, it has been shown that some nanomaterial additives can significantly improve lubrication performance by enhancing the thermal stability of the lubricant [6,7], all of which have a significant impact on fuel consumption. In addition, nanomaterials are relatively resistant to temperature, making them an interesting alternative or complement to traditional additives [8].

Many different metal oxide nanomaterials have been tested as lubricant additives [9,10], but zinc oxide (ZnO) has shown outstanding tribological performance, inducing a lower coefficient of friction and improving wear resistance [5,11,12]. In addition to tribological aspects, ZnO nanomaterials have delivered some antioxidant activity, especially acting as radical scavengers that break the propagation step of the lubricating oil oxidation mechanism [13,14].

However, the application of ZnO nanomaterials as a lubricant additive has been limited by their poor dispersibility in lubricating oils due to their high surface energy, causing agglomeration effects [15,16]. Therefore, it is necessary to improve ZnO dispersibility and stability in lubricants. There are different approaches to improve nanomaterials dispersibility in a fluid. Adding surfactants has become an effective and convenient method to modify the surface properties of nanomaterials and lower the surface tension of base fluids. However, the percentage of surfactant in nanolubricants should be carefully evaluated, since the viscosity of nanolubricants will be changed when surfactants are added [17]. One of the best methods to improve the dispersion and stability of the dispersions is to modify the surface properties of nanomaterials attaching surfactants on the surface of the NMs [18], as they provide steric repulsion, thus preventing agglomeration. Among them, oleic acid is one of the most commonly used surfactants, providing a chain length of around 2 nm, which is considered appropriate for nanomaterials with an average size of 10 nm; the polar head of the oleic acid attaches to the nanomaterial surface, whereas the non-polar chains cross-link with each other, allowing a stable dispersion of nanomaterials in the oil [19].

Some researchers have modified ZnO with organic coupling agents with the aim not only to improve its dispersibility, but also to take advantage of the surface protective capacity of these organic agents by generating a synergistic effect [17].

The review of the literature shows that there are very few studies using fully formulated engine oils [20], since they focus on lubricant baseoils [8,9,21,22], so the impact that functionalized ZnO nanomaterials have not only on tribological performance but also on how these metal oxide nanomaterials interact with other conventional antioxidants such as zinc dialkyldithiophosphates (often referred to as ZDDP) [23] and other types of additives in engine oils, is unclear.

Therefore, this study aims to evaluate the impact of the dispersion of zinc oxide nanomaterials functionalized on the surface with oleic acid, using a fully formulated engine oil and contributing to the advancement of knowledge in this area. To this end, dispersions have been prepared in the laboratory using different routes that have allowed us to perform a comparative study of (i) the dispersion of ZnO nanomaterial (20 nm) supported by the addition of oleic acid as a surfactant, (ii) ZnO functionalized (20 nm) with oleic acid with a degree of functionalization of 10%, and (iii) ZnO functionalized (20 nm) with oleic acid with a degree of functionalization of 80%. Each proposed route has produced nanolubricants with different cluster sizes and stability and, therefore, different physicochemical properties and tribological performances.

This study allows for the evaluation of the synergistic effect between oleic acid and ZnO nanomaterials in terms of physicochemical properties, oxidation stability, and tribological performance. Moreover, this study evaluates the influence of degree of functionalization and concentration on the overall performance of the lubricant.

## 2. Materials and Methods

### 2.1. Materials and Reagents

Commercial zinc oxide spherical nanomaterials in powder form with a primary particle size of 20 nm and a specific surface area of 50 m^2^/g (Iolitec, Heilbronn, Germany) were used.

Oleic acid (purity > 99%, Sigma-Aldrich, St. Louis, MO, USA) was used as a surfactant for the zinc oxide direct dispersion and as the functionalization group in the case of functionalized zinc oxide.

A reagent-grade n-hexane with a purity ≥ 99% (Sigma-Aldrich, St. Louis, MO, USA) was used as the liquid media to perform the zinc oxide functionalization.

In terms of the lubricant matrix, a synthetic high viscosity engine oil was selected. It is a fully synthetic oil with a mixture of Group-IV and Group-V baseoils, suitable for very high-performance engines, ensuring low friction and providing wear protection in engine rubbing surfaces. It has a density of 0.85 g/cm^3^ at 15 °C, a kinematic viscosity of 23 cSt at 100 °C and has a viscosity index of 167.

The lubricant has different primary antioxidants (radical scavengers) than aromatic amine antioxidants and phenolic antioxidants. It also contains secondary antioxidants (hydroperoxide decomposers) than molybdenum polysulfide complex and organophosphorus compounds group. Inductively Coupled Plasma Optical Emission Spectroscopy (ICP-OES, Ultima Expert, Horiba, Kyoto, Japan) analysis (standard ASTM D-5185 [24]) was performed on the lubricant to analyze the presence of additive elements, identifying Phosphorous (600–900 ppm), Zinc (500–900 ppm), and Molybdenum (40–100 ppm). 

### 2.2. Zinc Oxide Functionalization

**Figure 1:**
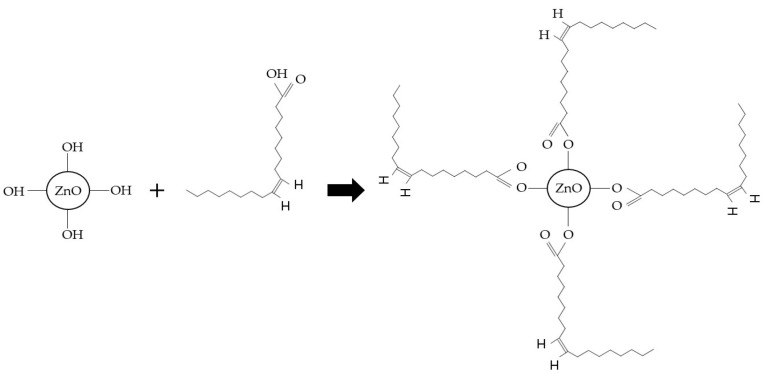
Zinc oxide functionalization mechanism.

The chemical anchoring of the oleic acid (OA) on the ZnO surface is accomplished by an esterification reaction between the carboxyl group (-COOH) of the OA and the hydroxyl group (-OH) present on the ZnO surface. Thus, the polar head of the oleic acid is oriented towards the nanomaterial, and the aliphatic (non-polar) chain is oriented towards the lubricant, which allows for the stabilization of the ZnO in the medium (Figure 1).

To begin, 4 mL of oleic acid was dissolved in 80 mL of n-hexane in a flask at 50 °C under stirring. Then, 1 g of the commercial ZnO nanomaterial was added to the above-mentioned solution and the mixture was sonicated with the aid of a Hielscher 1000 hdt ultrasonic tip (Teltow, Germany) for 5 min to effectively disaggregate the NMs and to ensure maximum interaction between the nanomaterial and the oleic acid group. The reaction mixture was kept under stirring for 90 min at 50 °C [25].

During the functionalization process agglomerates can form between the zinc oxide NMs, generating different populations of functionalized material, so that at the end of the reaction time the obtained mixture was subjected to different processes to obtain nanomaterials with different size ranges and with a lower polydispersity.

**Figure 2:**
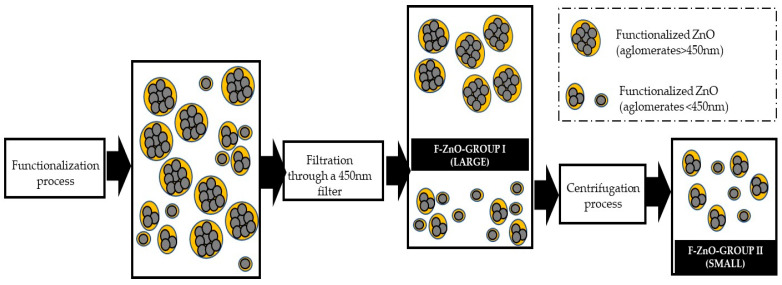
Process for obtaining small and large agglomerates of functionalized ZnO.

For this purpose, the sample was filtered through nitrocellulose with a pore size of 450 nm and compressed air up to 5–6 bar. The largest agglomerates (F-ZnO-Group I in Figure 2) were collected from the filter after washing with hexane and ethanol. The smallest agglomerates passed through the filter and were collected with the n-hexane and unreacted oleic acid. This mixture was centrifuged at high revolutions (1650 rpm, 20 min) to favor its decantation and underwent a multi-stage cleaning process with hexane for subsequent drying and obtaining the smaller agglomerates (F-ZnO-Group II in Figure 2).

### 2.3. Nanolubricants Preparation Protocol

Samples of nanolubricants (100 g) with different ZnO contents by weight (0.1 wt.%, 0.5 wt.%, and 1.0 wt.% with respect to the lubricant) were prepared according to the following two-step method.

In the case of functionalized ZnO, the amount of nanomaterial to be dispersed was weighed and added to the lubricant. The mixture was then homogenized using a disperser (Dispermat LC55, Reichshof, Germany) for 5 min at 2500 rpm and then ultrasonically dispersed for 10 min using a Hielscher UIP 1000 hdt ultrasonic tip (Teltow, Germany) at 60% amplitude. For non-functionalized nanomaterials, the protocol had an additional step prior to nanomaterial incorporation consisting of mixing the lubricant and oleic acid with the support of the disperser (5 min at 2500 rpm).

To avoid temperature increase during the ultrasonication operation, the samples were kept in a cold-water bath to dissipate the possible heat generated.

### 2.4. Characterization Techniques

#### 2.4.1. Functionalized Zinc Oxide Characterization

To evaluate the efficiency of the functionalization process, four analytical techniques were employed. Fourier transform infrared spectroscopy (FTIR) was used to verify the existence and type of chemical bonds. To determine the degree of functionalization, thermogravimetric analysis (TGA) and a carbon/sulfur analyzer were used. Finally, the morphology of the samples was observed using a scanning electron microscope (SEM).

FTIR spectra were obtained using a FT/IR 4700 (JASCO, Dunmow, UK) system to study the functional groups appearing in the functionalization reaction. Attenuated Total Reflectance (ATR) measurements were carried out from 400 to 4000 cm^−1^ and 30 scans were taken for each sample, with a resolution of 4 cm^−1^.

Thermobalance TGA/SDTA851e (Mettler Toledo, Columbus, OH, USA) was employed to evaluate the functionalization degree (FD). Above 200 °C, the mass loss was associated to the organic matter degradation (thus associated to the degree of functionalization with oleic acid). TGA analysis was performed in duplicate, with a heating rate of 10 °C/min, from 25 to 900 °C, in a nitrogen (N_2_) atmosphere and flow rate of 50 mL/min.

The carbon and sulfur analyzer (LECO CS-200 Leco Corporation, St. Joseph, MI, USA) was used to measure the percentage of carbon of the functionalized nanomaterials. To do so, a test sample is burnt in an induction furnace using 99.5% quality oxygen in the presence of an accelerator to obtain carbon dioxide and sulfur dioxide, which are quantified by infrared absorption. With the percentage carbon value of the sample obtained, the % of oleic acid was calculated (assuming that 76.5% of the total molecular weight of oleic acid is carbon). The degree of functionalization (in percentage) was then calculated as the average of the % of functionalization obtained by TGA and that obtained by the carbon and sulfur analyzer.

#### 2.4.2. Nanolubricant Dispersion Characterization

Laser diffraction (LD) technique was used to evaluate the particle size distribution (range 0.02–2000 µm) of nanofluids with a MasterSizer Hydro2000 (Malvern Instruments Worcestershire, UK). The measurement was performed by adding the nanofluid sample into the dispersion unit filled with hexane until achieving a laser light obscuration of 15%. The dispersion unit kept the mixture stirring at 2000 rpm. Each sample was measured three times with a 5 s delay between each measurement, and the result was reported as an average of the taken measurements. 

The sedimentation stability under static storage of the formulated nanolubricants was analyzed through visual observation of the samples over time (just after preparation, 72 h, 11 days, and 25 days). Thus, the time was determined at which the sample was stable by visual inspection.

#### 2.4.3. Physic Chemical Characterization of Nanolubricants

The thermo-oxidative stability of the different samples was evaluated by determining the Oxidation Induction Time (OIT), which is a relative measure of the resistance of a material to oxidative decomposition. The OIT was measured by means of High-Pressure Differential Scanning Calorimetry (HPDSC1/130 Mettler Toledo, Columbus, OH, USA) according to standard ASTM D-6186 [26], heating 7 mg of sample (in an aluminium crucible) to 207 °C at a heating rate of 10 °C/min and keeping it isothermally at 207 °C for 2 h under 35 bars of pressure at a constant oxygen flow of 100 mL/min. The onset time of the oxidation reaction was taken as the intersection of the extrapolated baseline and the tangent line (leading edge) of the exotherm.

Viscosity is another one of the parameters that was analyzed in order to assess whether the presence of nanomaterials can affect the rheological behavior of the lubricating oil. For this purpose, the flow curve (viscosity vs. shear rate) of the reference oil and the nanolubricants was evaluated. The dynamic viscosity measurements were carried out with the Discovery Hybrid Rheometer 2 (DHR-2 TA instrument, Delaware, USA). The tests were performed with a 40 mm plate applying a shear rate from 0.01 s^−1^ to 1000 s^−1^. Viscosity was measured at 40 °C and 100 °C.

The measurements of density were performed in an electronic densimeter DX4 equipped with RM40 Refractometer (Mettler Toledo, Columbus, OH, USA) at 40 °C and 100 °C of temperature.

Viscosity index (VI) was also calculated following the standard ASTM D-2270 [27]. This parameter is an arbitrary, unitless measure of the change in viscosity of a fluid in relation to the change in temperature. A higher VI is more desirable because it enables the lubricant to provide a more stable lubricating film over a wider temperature range. The viscosity index was calculated using kinematic viscosity values determined by the ratio of dynamic viscosity to density.

#### 2.4.4. Tribological Characterization of Nanolubricants

**Figure 3:**
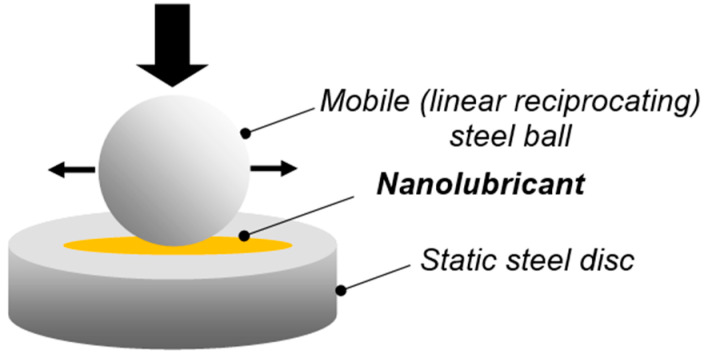
“Ball on disc” test configuration.

A steel–steel contact under pure sliding conditions was selected to determine the friction and antiwear properties of the nanolubricants. In particular, tests were conducted using a “ball on disc” configuration with linear reciprocating motion (SRV tribometer, Optimol Instruments, Munich, Germany) according to ASTM D-5707 standard [28]. A 10 mm diameter mobile ball was pressed against a stationary disc at a constant normal load (20 N) and then slid at a fixed frequency (5 Hz) and amplitude (1 mm) for 30 min (see scheme in Figure 3). AISI 52100 was the material for both test specimens, having an arithmetic average roughness (Ra) of 0.025 and 0.050 µm, respectively. A controlled volume of the samples (0.3 mL) was placed on the surface of the disc and heated to 80 °C. During the test, the evolution of the coefficient of friction (CoF) was continuously recorded. At the completion of the testing time, the wear mechanisms occurring on the contacting surfaces were studied by Optical microscopy (DM 2500 MH, Leica Microsystems CMS, Mannheim, Germany) measuring the ball-wear scar diameter and more in detail by using a Scanning Electron Microscope (Ultra Gemini-II Carl Zeiss SMT, Oberkochen, Germany). Two test runs per lubricant were carried out.

## 3. Results and Discussion

### 3.1. Zinc Oxide Nanomaterial Functionalization (OA-ZnO)

**Figure 4:**
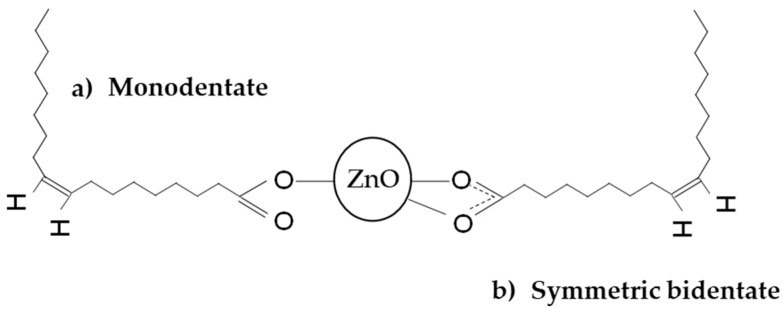
Types of functionalization proposed for the ZnO with OA.

The effective surface functionalization of ZnO with oleic acid (OA) was evaluated by FTIR, TGA, and LECO. Different types of covalent anchorage such as monodentate binding mode or bidentate binding mode can be distinguished (Figure 4), as has been detailed before by several researchers [29,30,31].

**Figure 5:**
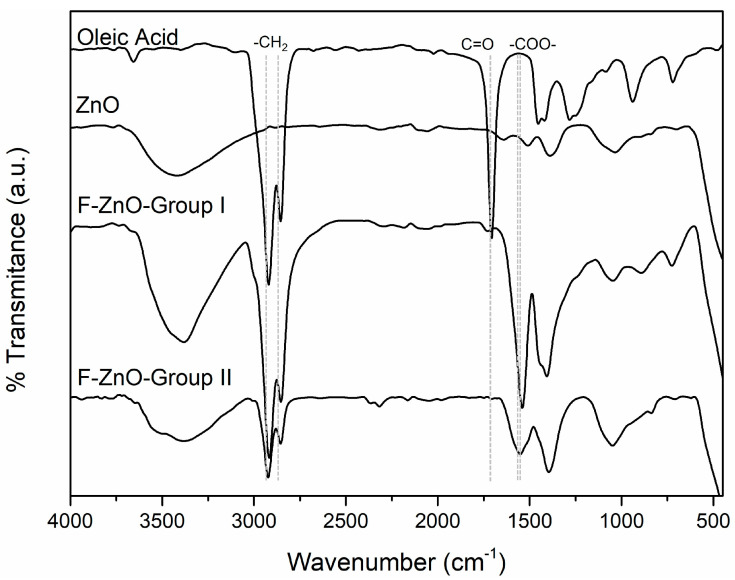
FTIR spectra of oleic acid, ZnO, functionalized F-ZnO-Group I, and F-ZnO-Group II.

Figure 5 shows the FTIR spectra of oleic acid, ZnO, and the functionalized ZnO nanomaterials (F-ZnO-Group I, F-ZnO-Group II). The FTIR spectrum of oleic acid showed the asymmetric and symmetric stretching vibrations of -CH_2_, -CH_3_ at 2920 cm^−1^, and 2855 cm^−1^, respectively, and the C-H bending vibration band appeared at 1443 cm^−1^. The intense band observed at 1707 cm^−1^ was attributed to asymmetric -C=O stretch.

The spectrum of ZnO nanoparticles showed two main peaks, the absorption band at 3410 cm^−1^, corresponding to the stretching vibrations of the O-H group attributed to the presence of hydroxyl groups and the vibration band observed at 468 cm^−1^, which corresponds to the stretching vibration of the zincite structure (Zn-O) [32].

The functionalized ZnO spectrums (ZnO-OA) presented the absorption band at 3410 cm^−1^, (stretching vibrations of the O-H group) attributed to the presence of hydroxyl residue. In addition, the C-H absorption bands are retained in OA-modified ZnO nanomaterials. The peaks that appear at 1543 and 1435 cm^−1^ were assigned to the asymmetric and symmetric stretching vibrations of -COO-, respectively, which indicates the presence of bidentate carboxylate bonding. It is worth mentioning that the FTIR spectrum seen in Figure 5 was very similar to that reported by Mariño et al. [20] for other ZnO nanomaterials functionalized with oleic acid.

On the other hand, to know the degree of functionalization, thermogravimetric analysis (TGA) and sulfur and carbon analyzer were used.

Table 1 presents the results of functionalization degree (FD) obtained from TGA analysis (mass loss from 200 °C to 900 °C) and carbon/sulfur analysis (by calculating it from the % C obtained). On the last column the average of FD was calculated, as both results were in a good agreement.

**Figure 6:**
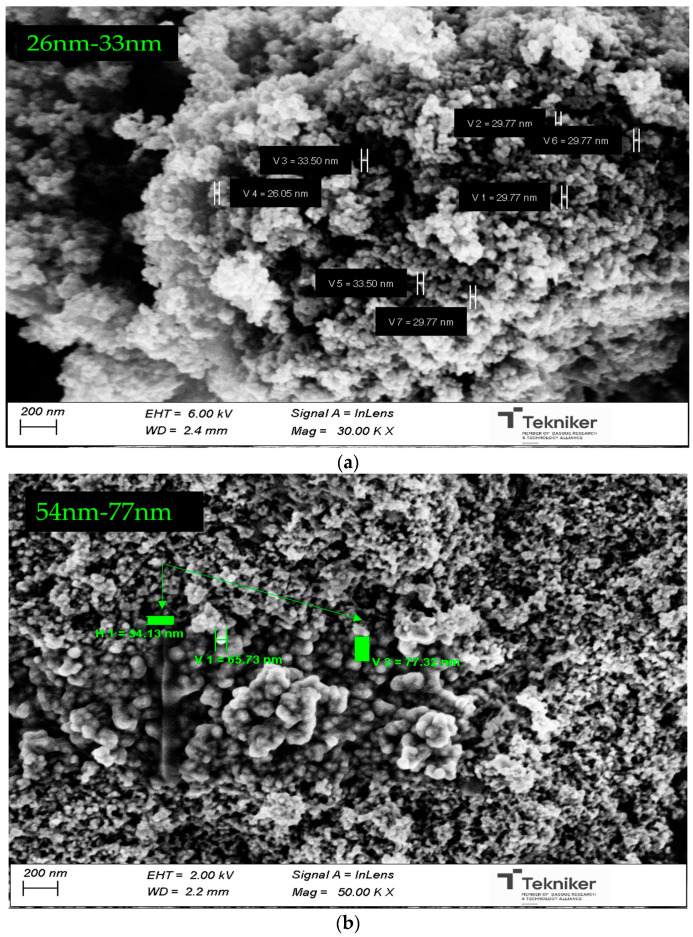

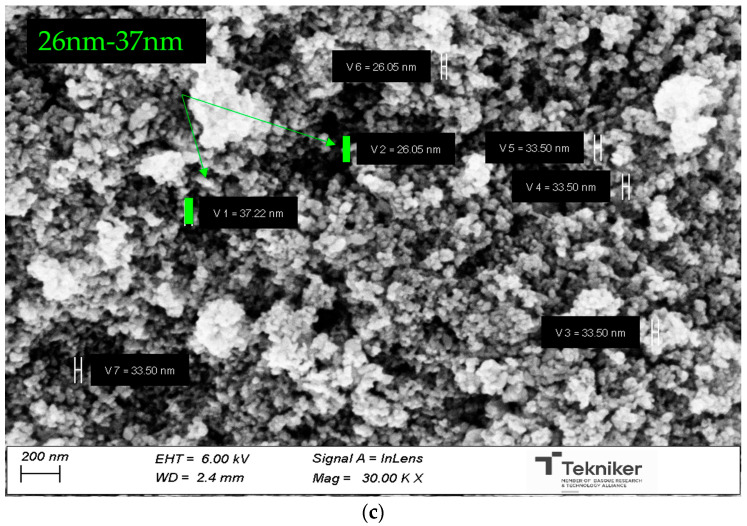
Scanning electron micrographs of the raw and modified ZnO nanomaterials (**a**) Raw ZnO (26–33 nm), (**b**) F-ZnO-Group I (54–77 nm), and (**c**) F-ZnO-Group II (26–37 nm).

SEM analysis of commercial ZnO reveals that the nanomaterials were nearly spherical in shape, with a primary particle diameter between 26 and 33 nm (Figure 6a). For the functionalized nanoparticles, F-ZnO-Group I (Figure 6b) and F-ZnO-Group II (Figure 6c), the primary size hardly varied with respect to non-functionalized ZnO, showing a primary particle diameter between 23–37 nm and 54–77 nm, respectively. SEM images can visualize the NM agglomeration.

### 3.2. Nanolubricants Dispersion Analysis

**Figure 7:**
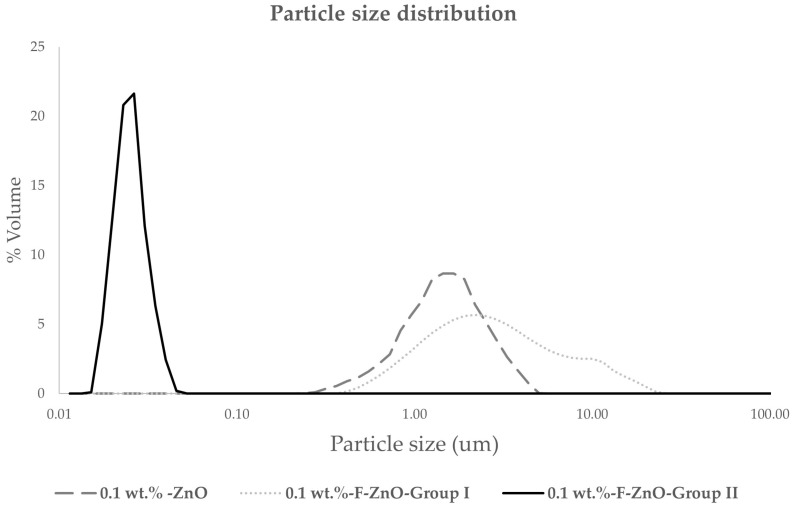
Particle size distribution of formulated systems 0.1 wt.% of ZnO via surfactant addition, 0.1 wt.% of F-ZnO Group I, and 0.1 wt.% of F-ZnO Group II.

Particle size distribution (PSD) (Figure 7) of the nanolubricants formulated at 0.1 wt.% of ZnO was measured to verify the cluster size promoted by each type of nanoparticle. Although the primary particle size of the different ZnO was similar, as it was identified by SEM, the proposed dispersion technique (surfactant, or surface functionalization) has conditioned the size of the obtained clusters, which was related not only to the stability of the systems but also to the promoted properties.

Laser diffraction measurements reveal that functionalized ZnO-Group II promoted the lower mean cluster particle size (d(0.5) of 0.02 µm), while ZnO-Group I and ZnO provided samples with similar and higher cluster particle sizes (d(0.5) of 2.88 µm and 1.95 µm, respectively) (Table 2).

The visual appearance is a key aspect for a lubricant, since for the automotive sector appearance, colour, and turbidity can be important criteria for lubricants and are usually the aspects that show the quality of the fluid, and a turbid aspect may indicate an incompatibility within the components of the formulation, with a potential impact in the performance. Therefore, when formulating a nanolubricant, it is a key aspect to minimize any alterations in the visual appearance of the lubricant. The aspects of nanolubricants are shown in Table 3.

**Figure 8:**
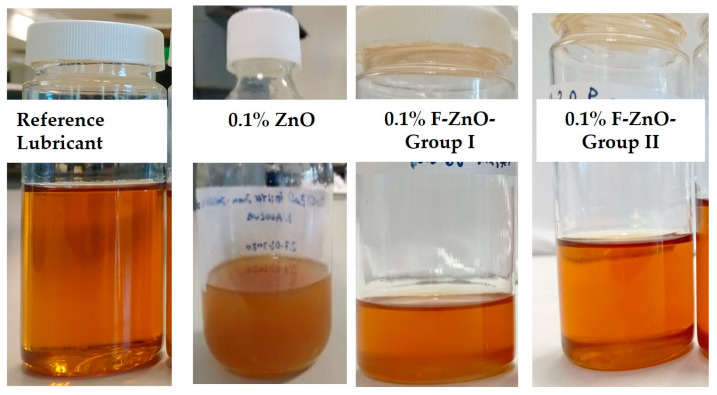
Visual appearance of raw lubricant and formulated nanolubricants at 0.1wt.%.

While formulations based on functionalized F-ZnO-Group I or by adding ZnO promoted a cloudy appearance, samples formulated with F-ZnO-Group II showed a translucent appearance similar to that of the original lubricant, resulting in a better formulated nanolubricant (Figure 8). Nanolubricant stability was evaluated by a visual analysis of the samples. Sample aspect was evaluated after preparation as well as after 25 days of storage under static conditions at room temperature, and it was found that after 25 days those samples based on F-ZnO-Group I or by adding ZnO showed signs of instability, thus increasing sample turbidity, while systems formulated with the higher functionalization degree in the lower cluster size, F-ZnO-Group II, did not change the sample aspect, evidencing that for an effective dispersion, the lower cluster size combined with a high functionalization degree is more effective to keep electrostatic repulsion.

### 3.3. Nanolubricants Physic Chemical Properties

From the measurements obtained in the HPDSC, the oxidation induction time (OIT) of each system was determined for comparison with that of the reference lubricating oil. The longer OIT refers to the extended oxidative stability of oil.

**Figure 9:**
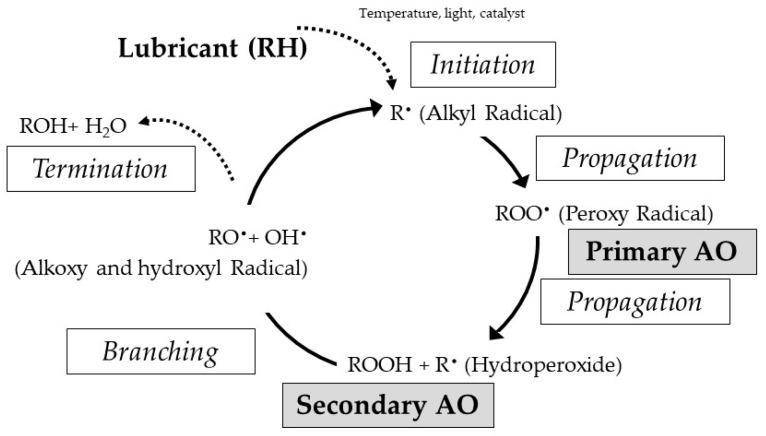
Lubricating oil degradation mechanism and stages in which the different antioxidants (A) act.

The oxidation of lubricating oils takes place in the base fluid via a free radical mechanism, which has been explained in detail in the literature. The mechanism is divided into four steps: Initiation, Propagation, Branching, and Termination (see Figure 9). There exist different alternatives to inhibit the oxidation mechanism, with one of them being the use of radical scavengers. Radical scavengers are referred to as primary antioxidants (AO), since they function by breaking the Propagation Step, thus interrupting the free radical chain growth [33].

Table 4 shows the obtained values for the Oxidation Induction Time (OIT) of the prepared samples. Additionally, the OIT of the lubricant with oleic acid (without NM) was also measured in order to evaluate the effect that the surfactant has on the OIT of the reference.

ZnO nanomaterials are well-known (n-type) semiconductors, having antioxidant activity due to the transfer of electron density located at oxygen, and exhibit antioxidant activity especially for scavenging free radicals, thus providing protection against damage caused by free radicals, which are highly reactive species that can cause oxidative stress [34]. However, it is important to note that although ZnO nanomaterials have shown antioxidant activity, their effect can vary depending on factors such as size, surface properties, and concentration [14,35].

While ZnO nanomaterials could act as antioxidant additives, fatty acids such as oleic acid are known to show low oxidative–thermal stability, this being one the main drawbacks of using these compounds [34,36,37] as lubricants.

The OIT of the reference lubricant was recorded to be about 49.33 min. Oleic acid added into the reference lubricant negatively affected its stability (OIT of 46.05 min), while the nanolubricants formulated with the F-ZnO-Group II nanomaterials showed an increase in the OIT compared with the reference; thus, the nanolubricant containing 1.0 wt.% was the one providing the highest OIT (an increase of 19.80%).

Experimental results showed that the impact on thermo-oxidative stability depends on the type of ZnO material used and the material concentration. Thus, the dispersion of ZnO with oleic acid as surfactant as well as functionalized F-ZnO-Group I NM, where the cluster size is 1.95 and 2.88 µm, respectively, promoted an OIT decrement. The dispersion of F-ZnO-Group II at cluster size 0.02 µm significantly enhances the thermo-oxidative stability of the lubricant.

These results agree with previous reports, where the higher antioxidant capacity was found for the smaller particles with a higher bandgap [35]. This was attributed to the fact that the smaller the particle size was, the greater the surface area, and therefore, the greater the number of active sites on the surface of the ZnO [14]. The role proposed for oleic acid combined with ZnO nanomaterials was a nanomaterial surface activator [20]. The combined effect of surfactant and nanomaterials has shown good antioxidant properties.

Viscosity is a property occurring due to the internal frictional force between different layers of fluids as they move relative to each other. The suspended nanomaterials in the oil raised its viscosity as a result of the collisions between the nanomaterials and the base fluid, but the influence of nanomaterials on lubricant rheological properties is still unclear.

**Figure 10:**
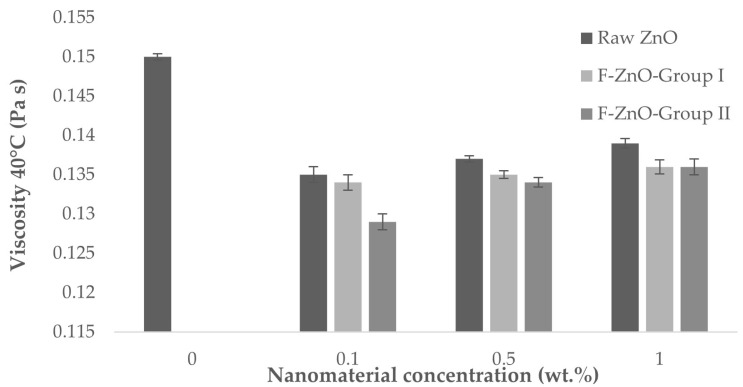
Viscosity of nanolubricants measured at 40 °C.

Experimental results (Table 5) show that, in this study, the viscosity in all the cases was reduced at 40 °C with the addition of nanomaterials (Figure 10). Apart from this, the addition of surfactant also causes a decrease in the viscosity of the lubricant. The viscosity of the formulated nanolubricants decreased compared to the pure lubricant not only because of the addition of surfactant, but also because the nanomaterials can act as ball bearings, reducing the friction between the moving surfaces and as a result less energy is required to move the surfaces, which can lead to a reduction in viscosity [38]. This experimental result is in agreement with previous results reported in the literature [39], in which nanomaterials are proposed as viscosity modifiers due to their capacity to reduce oil viscosity.

It can be observed that the obtained viscosity values agree with the generally observed behavior, i.e., viscosity depends on three main aspects, nanoparticle fraction dispersion, nanomaterials agglomeration, and thus, nanomaterials dispersibility and temperature. Three tested systems showed a slight viscosity increment as the concentration of nanoparticle increased; when nanomaterials are added to the base fluid, the van der Waals force between the nanomaterials and the base fluid causes agglomeration of the nanomaterials, preventing the movement of the base fluid’s molecules, which results in increased viscosity [40,41].

Moreover, the cluster size in which the nanoparticle is dispersed will affect the viscosity value, as large agglomerates show a lower Brownian motion and an increase in the internal shear stress in the nanolubricant, and therefore an increase in the viscosity [40,42]. A good dispersion of functionalized nanoparticles can lead to a decrease in lubricant viscosity due to the compatibility between the surface organic chains of the nanomaterials and the lubricant, results obtained for the F-ZnO Group II.

As the temperature increases, the viscosities of both pure lubricants and nanolubricants exhibit a predictable pattern of diminishing [43], which is commonly observed behavior in lubricants. The rise in temperature aids particles in overcoming Van der Waals attractive forces, leading to the disintegration of nanoparticle clusters suspended within the base fluid. Consequently, the intermolecular interactions between molecules weaken, resulting in a noticeable reduction in viscosity. In the case study where all samples were measured at 100 °C, comparable results were observed.

It can be also observed that the viscosity index increased, improving the performance of the engine oil over different temperature conditions, which is an important feature for a high quality engine oil [44].

Regarding experimental data of densities obtained for prepared nanolubricants, a slight and depreciable modification of the proposed nanomaterial loads has been observed.

### 3.4. Tribological Assesment

Friction and wear tests were carried out with the reference and obtained nanolubricants. Two additional lubricant samples were prepared in order to investigate the tribological effect of the addition of oleic acid in the reference oil.

**Figure 11:**
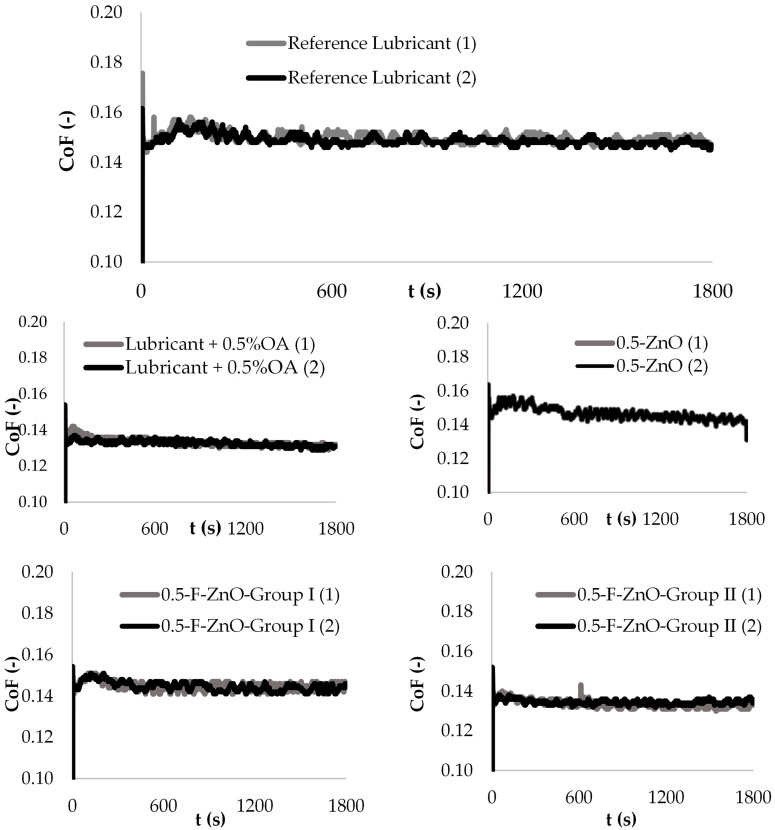
CoF vs. time for the reference and samples of reference with 0.5 wt.% of AO and nanolubricants with 0.5 wt.% content of nanomaterials. (1) and (2) refer to different test runs with the same lubricant.

All the nanolubricants provided a very stable frictional behavior equivalent to the one observed with the reference lubricant. Figure 11 shows results obtained with the reference lubricant and some representative results, in particular those containing 0.5 wt.% of tested nanomaterials and 0.5 wt.% of oleic acid added into the lubricant. These observations suggest that dispersed nanomaterials remain homogeneously dispersed through time in the base oil and no substantial agglomeration of nanoparticles occurred in the contact area, which could have led to friction instabilities.

For each test run, an averaged value of the coefficient of friction (CoF) was calculated. A main value and its standard deviation were later calculated considering the two test runs. Finally, the relative reduction in the CoF achieved by the nanolubricants in comparison with the reference value, i.e., the CoF obtained with the reference lubricant, was also calculated (Table 6).

As a first approach, the results confirm that a larger amount of oleic acid caused a further decrease in the CoF. In particular, a CoF of 0.120 was obtained with a 1.5 wt.% concentration of oleic acid, compared to the reference value of 0.149. This behavior is explained by the absorption of the additive molecule on the steel surfaces, which leads to a lower friction coefficient, and thus, a higher additive concentration [45].

**Figure 12:**
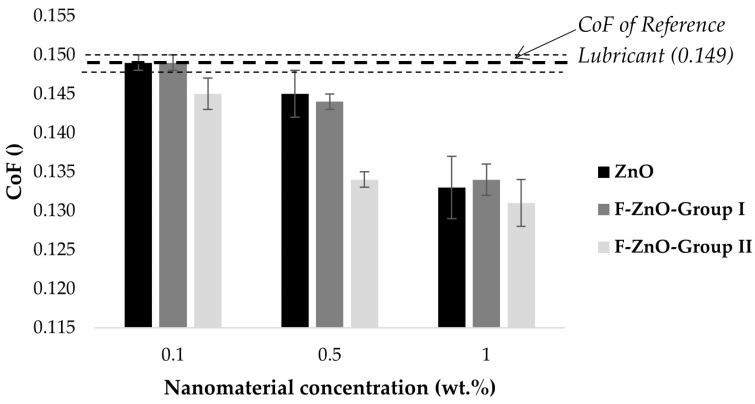
Mean coefficient of friction of nanolubricants with different nanomaterial types and concentrations.

Focusing on the effect of the nanomaterial concentration (Table 6 and Figure 12), it was observed that, for these nanomaterials, the addition of 0.1 wt.% did not provide any CoF improvement. However, higher concentrations (0.5 wt.% and 1 wt.%) caused more significative CoF reductions in the range of 3–10% and 10–12%, respectively. In particular, it was observed that, in all the cases, having 1% of nanomaterials implied the most noticeable reduction in the CoF compared to the reference lubricant. This reduction was predictably caused by different mechanisms previously reported in the literature: (i) ZnO has been proven to show good friction and antiwear performance, promoting relative slippage of the nanomaterials in both counterparts [20,41] and (ii) oleic acid produces a molecular film adhering to the surface by physical or chemical absorption, reducing the CoF [16,46,47,48].

In addition, it was observed that the CoF reduction was similar to non-functionalized particles (in which OA is simply added as a surfactant) and with the functionalized F-ZnO-Group I (see Figure 11). This fact can be due to the synergetic effect of the low functionalization degree of the F-ZnO-Group I (9.8%), jointly with a more efficient cluster size distribution which could promote a rolling protective mechanism.

However, in the case of the more functionalized nanomaterials (F-ZnO-Group II), a greater reduction in the coefficient of friction was observed. This reduction was appreciated even at a lower concentration of nanomaterials (2.68% of reduction at 0.1 wt.%) and the effect was enhanced at higher concentrations (CoF reduction of 10% and 12% for 0.5 wt.% and 1.0 wt.%, respectively). This noticeable improvement with the F-ZnO-Group II can be justified for different reasons:

(i) The functionalization degree of the F-ZnO-Group II was 86.9%, so the oleic acid content was higher than for the other cases and, as previously proven, the oleic acid reduced the CoF;
(ii) The smallest size of the nanoparticle cluster was in the same range as the surface roughness, which can suggest that the average size of the valleys on the surface could be filled, leading to friction and wear reduction. These results supported the proposed surface mending mechanism of the particle-based additive function [49];
(iii) The oleic acid promoted the dispersity of ZnO in the lubricant system and the random deposit probability of ZnO nanoparticles on the rubbing surface, acting as a rolling bearing [5].

**Figure 13:**
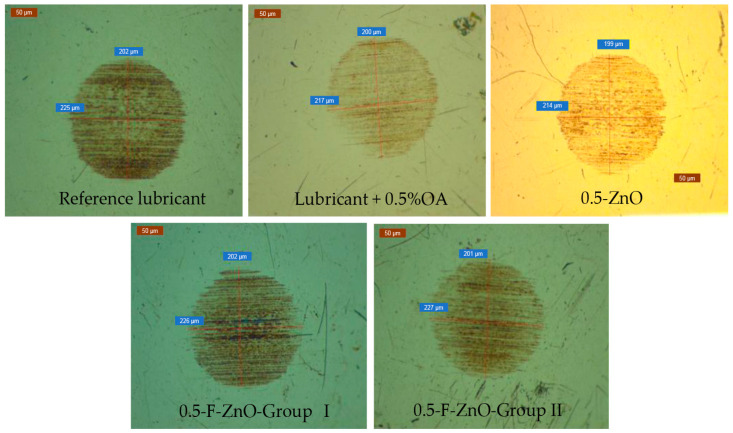
Appearance of ball wear scars for the reference and nanolubricants with 0.5 wt.% content of nanomaterials or oleic acid.

In relation to the capability of nanolubricants to protect surfaces against wear, all the systems promoted wear reduction in comparison to the reference oil (see Table 7, and Figure 13). Nevertheless, these improvements were less relevant than those observed in frictional behavior, reaching a maximum reduction of 6% with a 1 wt.% of functionalized nanomaterials. This maximum reduction effect was obtained with F-ZnO-Group I and F-ZnO-Group II.

**Figure 14:**
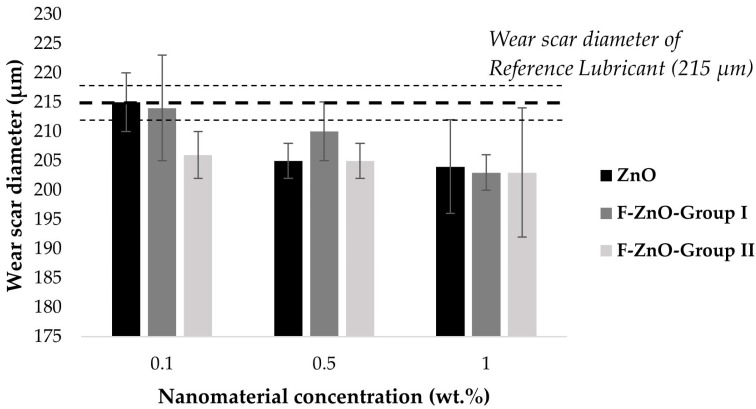
Wear scar diameter for the nanolubricants at different concentrations.

It was noteworthy that, for the functionalized F-ZnO-Group II nanomaterials, an improvement was observed even at the lowest concentration (0.1 wt.%) (Figure 14).

Once it was observed that the best tribological behavior was obtained with functionalized ZnO nanomaterials with a high degree of functionalization and dispersed in the lower cluster size (F-ZnO-Group II), nanolubricants with different amounts of nanomaterial and reaching higher concentrations (till 2 wt.%) were prepared to find the limits of use of these nanoadditives in the application of the studied system.

**Figure 15:**
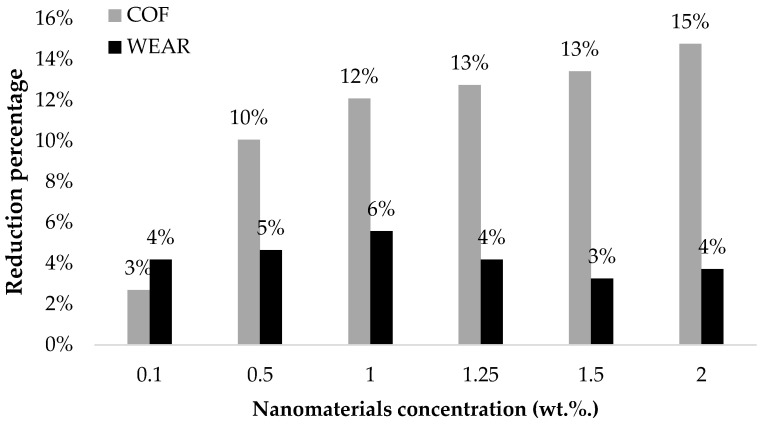
CoF and wear scar diameter for the prepared nanolubricants at different concentrations with nanomaterial F-ZnO-Group II.

On the one hand, the results (Table 8, Figure 15) revealed a clear reduction in the mean CoF with the addition of F-ZnO-Group II nanomaterials. In particular, the higher the content of the nanomaterials was, the lower the CoF. Thus, the most noticeable result was obtained with a 2 wt.% concentration (CoF = 0.127), which provided a reduction of 15% of the CoF in comparison with the reference lubricant (mean CoF = 0.149). Again, a smooth, stable, and highly reproducible behavior of the coefficient of friction through time was observed for this nanolubricant. In addition, it must be mentioned that, even with a low concentration of nanomaterials, i.e., 0.5 wt.%, a significant reduction of 10% in wear scars was also achieved. On the other hand, with regards to wear protection, no clear correlation between particle content and ball wear diameter was observed.

**Figure 16:**
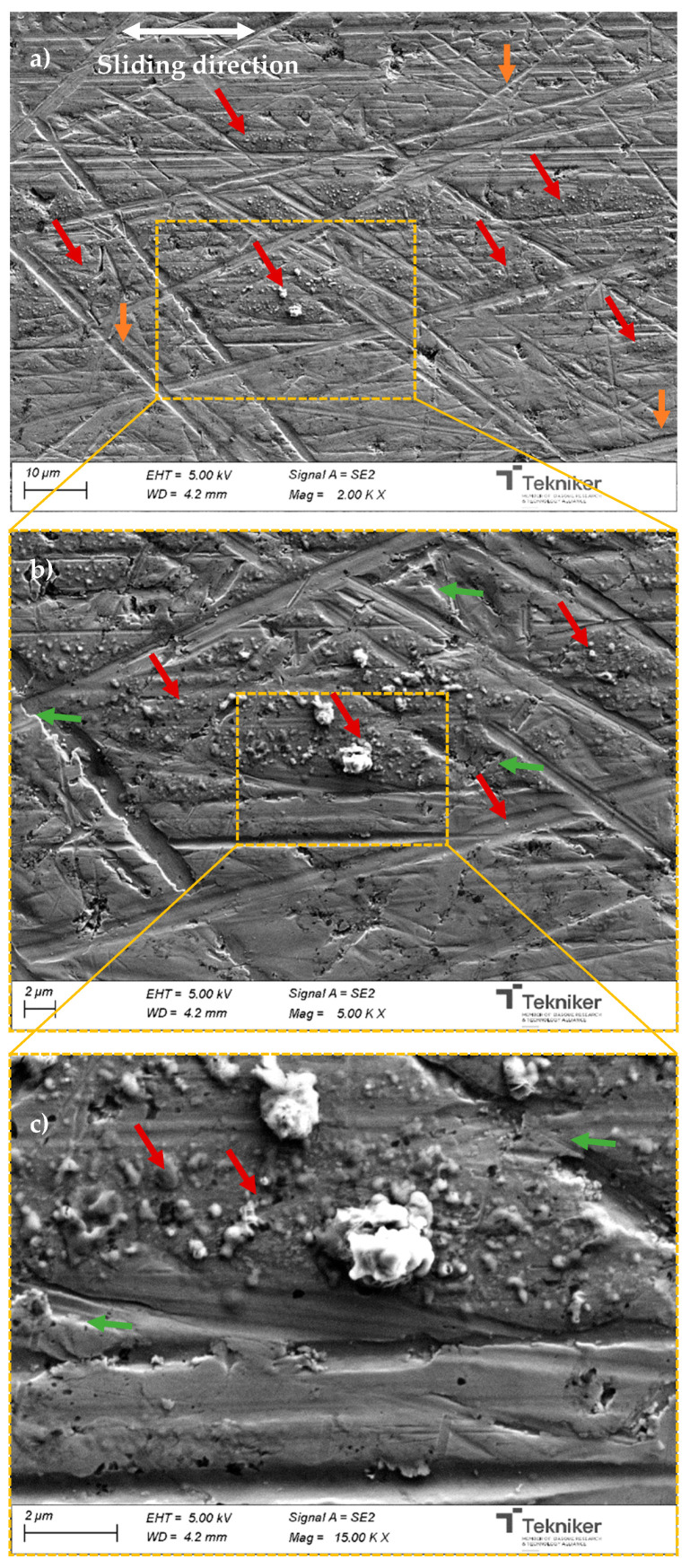
(**a**–**c**) Scanning electron micrographs of the disc wear scars tested with a nanolubricant containing a 2 wt.% of F-ZnO-Group II. Arrows indicate examples of different observations. White arrows: sliding direction; red arrows: ZnO nanomaterials on the wear track; orange arrows: original finishing mark from the disc lapping; green arrows: adhesive wear.

Once it was demonstrated that the best tribological behavior was obtained with 2 wt.% of F-ZnO-Group II nanomaterials, a dedicated analysis of the disc wear scars was carried out to investigate the wear mechanism promoted by the nanomaterials (see Figure 16).

It is known that nanomaterials may contribute to lubrication through several mechanisms such as rolling, mending, or tribofilm formation [5,50]. Evidence was found of the presence of a significant quantity of nanomaterials evenly distributed on the wear scar area, in some cases as individual nanomaterials and in others as agglomeration-formed particles of a bigger size. These nanomaterials kept their original spherical shape, which denoted that the primary involvement of a protective mechanism that is related to the well-known bearing effect. The rolling mechanism is enhanced if the number of “ball bearings” in the contact is increased, which correlated with the observed results; a higher concentration of nanomaterials led to a higher friction decrease. This observation is in accordance with other authors [9,51]. Additionally, it was observed that the steel surface was smoothed as a consequence of the severe contact conditions, which promoted locally adhesive wear on the disc.

**Figure 17:**
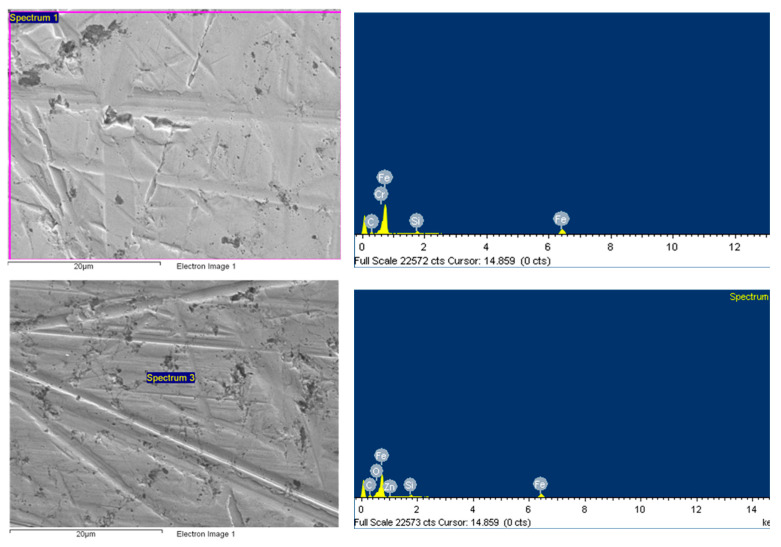

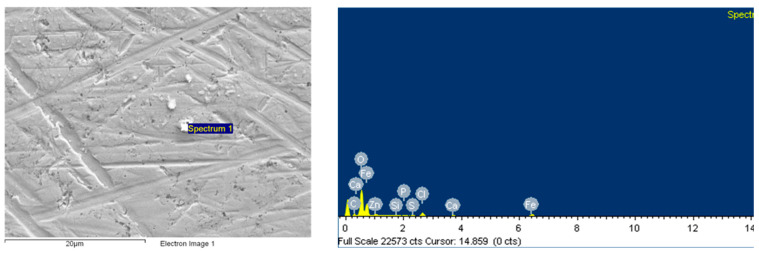
SEM-EDS spectrums on the disc of (up to down) non-tested steel surface, on wear scar with reference lubricants, and on wear scar with nanolubricant containing a 2 wt.% of F-ZnO-Group II.

Additional characterizations were conducted to analyze the possibility of having a tribofilm on the wear track. SEM-EDS (energy dispersive X-ray spectroscopy) analysis was performed, although it is well-know that EDS spectra includes information on elements below the surface (1 µm depth approximately). However, the approximate values of the element content concentration can be used for qualitative analysis. Actually, spectra done in different steel discs and zones (Figure 17) and element content (Table 9) confirmed the formation of a tribofilm, but, in this case, due to the base oil additives, which was confirmed by the detection of Zn, P, and S. These elements are directly related to the ZDDP and detergents commonly used in engine oils.

On top of this, the presence of functionalized ZnO nanomaterials was confirmed by the noticeable increase in O and the presence of Zn in the spectrum of the analyzed nanoparticle in the test carried out with this nanolubricant.

Since the nanoparticles were a zinc oxide, the base oil had Zn in its composition and there was no evidence of nanoparticle breakage, thus, it was not possible to confirm if the characterization technique used in this study had any nanoparticle mending effects.

## 4. Conclusions

Three different ZnO nanomaterials, (i) ZnO nanomaterial measuring 20 nm, (ii) Functionalized ZnO (20 nm) with oleic acid with a functionalization degree of 10% (F-ZnO-Group I), and (iii) Functionalized ZnO (20 nm) with oleic acid with a functionalization degree of 80% (F-ZnO-Group II), have been dispersed at different concentrations in a fully formulated engine oil to analyse the visual aspect, thermo-oxidative stability, viscosity, and tribological performance.

In a first evaluation, dispersions prepared at 0.1 wt.%, 0.5 wt.% and 1 wt.% with each ZnO nanomaterial were compared. This study has allowed us to evaluate the existing synergistic effect between oleic acid and ZnO nanomaterials and how the dispersion strategy determines the performance of the lubricants. Oleic acid has been able to improve the dispersibility of the ZnO nanomaterials, with the most effective route being surface functionalisation versus its role as a surfactant. The size of the functionalised and dispersed ZnO clusters affected all evaluated properties and the performance of the engine oil. In particular:

The smaller the ZnO cluster with a higher functionalisation degree (F-ZnO-Group II), the higher the stability of the system (more than 25 days of storage). Meanwhile, systems with a higher cluster size have shown lower stability as well as promoting sample turbidity.

The F-ZnO-Group II-dispersed nanomaterials showed an increase in the OIT compared to the reference, while dispersion formulated with other nanomaterials were worse regarding this property when compared with the reference. A direct relationship was found between the increment in the OIT and F-ZnO-Group II content and, i.e., the nanolubricant containing 1.0 wt.%, providing the highest OIT (an increase of 19.80%). This is due to the role of ZnO as an antioxidant (electron scavenger mechanism) whose activity is enhanced when dispersed at a lower size. The smaller the particle size, the greater the surface area and the number of active sites on the ZnO surface. A relationship has also been found between nanoparticle loading and the benefits found.

Rheological properties are aligned with the general observed behavior, i.e., viscosity depends on three main aspects, nanoparticle fraction dispersal, nanomaterials dispersibility, and temperature. Tested systems have shown a slight viscosity increment as the concentration of nanoparticle increases; and decreases with the temperature.

Moreover, the cluster size in which the nanomaterial is dispersed has affected the viscosity value, as large agglomerates of dispersed ZnO has increased the viscosity. It has been found that a good dispersion of NMs with a functionalization that promotes the compatibility between the nanomaterials and the lubricant can lead to a decrease in viscosity.

Furthermore, in terms of tribological properties, non-functionalized ZnO and F-ZnO-Group I provide similar CoF reduction behaviour, being negligible for 0.1 wt.%, and causing CoF reductions around 3% and 10% for additions of 0.5 wt.% and 1 wt.%, respectively. Systems based on F-ZnO-Group II have reduced the CoF up to 12% for 1 wt.%.

From the first evaluation in which three ZnO nanomaterials were tested at different concentration up to 1 wt.%, Those systems prepared with the F-ZnO-Group II nanomaterials have promoted better results mainly due to the accurate selection of NM and proposed dispersion technique that promotes high NM stability in a lower cluster size.

In a second evaluation, the tribological performance of the F-ZnO-Group II systems prepared from 0.1 wt.% up to 2 wt.% have been compared, and the 2 wt.% F-ZnO-Group II was the sample that showed a significant tribological improvement compared to the rest (15% of COF reduction), presumably due to the synergy between cluster size and degree of functionalisation. Tribological protection effects can be exposed by three mechanisms, and by achieving ZnO clusters in the roughness range, these can fill in the roughness by smoothing the surface, giving rise to the well-known filling mechanism secondly, because the spherical NMs can act as bearings to improve sliding on the surface; and thirdly, because the oleic acid also acts as a friction coefficient enhancer.

## Figures and Tables

**Table 1:** Results of functionalization degree for the different nanomaterials.

Sample	% FD (TGA)	% C (Carbon/Sulfur Analysis)	% FD (Carbon/Sulfur Analysis)	% FD
F-ZnO-Group I	9.5 ± 0.1	7.6 ± 0.05	10.0	9.8 ± 0.1
F-ZnO-Group II	84.5 ± 0.4	68.3 ± 0.06	89.2	86.9 ± 0.4

**Table 2:** Mean particle size d(0.5) of systems formulated at 0.1% in weight.

Sample	d(0.5) µm
0.1-ZnO	1.95
0.1-F-ZnO-Group I	2.88
0.1-F-ZnO-Group II	0.02

**Table 3:** Aspect of nanolubricants.

Sample	Aspect
Reference Lubricant	Translucent aspect
Lubricant + 1%OA	Translucent aspect
0.1-ZnO	Turbid aspect
0.5-ZnO	Turbid aspect
1.0-ZnO	Turbid aspect
0.1-F-ZnO-Group I	Slightly turbid aspect
0.5-F-ZnO-Group I	Slightly turbid aspect
1.0-F-ZnO-Group I	Slightly turbid aspect
0.1-F-ZnO-Group II	Translucent aspect
0.5-F-ZnO-Group II	Translucent aspect
1.0-F-ZnO-Group II	Translucent aspect

**Table 4:** OIT of reference and formulated systems.

Sample	OIT (min)	% Variation
Reference Lubricant	49 ± 0.5	-
Lubricant + 1%OA	46 ± 0.3	−6
0.1-ZnO	43 ± 0.7	−12
0.5-ZnO	49 ± 0.7	−1.2
1.0-ZnO	50 ± 0.5	1
0.1-F-ZnO-Group I	45 ± 0.4	−9
0.5-F-ZnO-Group I	47 ± 0.6	−5
1.0-F-ZnO-Group I	50 ± 0.5	0.5
0.1-F-ZnO-Group II	50 ± 0.5	2
0.5-F-ZnO-Group II	55 ± 0.5	12
1.0-F-ZnO-Group II	59 ± 0.5	20

**Table 5:** Dynamic viscosity of systems.

Sample	Viscosity 40 °C (Pa s)	Viscosity 100 °C (Pa s)	Viscosity Index
Reference Lubricant	0.150 ± 0.0004	0.024 ± 0.0004	167
Lubricant + 1%OA	0.142 ± 0.0006	0.024 ± 0.0006	171
0.1-ZnO	0.135 ± 0.001	0.023 ± 0.0005	180
0.5-ZnO	0.137 ± 0.0004	0.024 ± 0.0007	177
1.0-ZnO	0.139 ± 0.0006	0.024 ± 0.0003	174
0.1-F-ZnO-Group I	0.134 ± 0.001	0.023 ± 0.0005	180
0.5-F-ZnO-Group I	0.135 ± 0.0005	0.024 ± 0.0006	179
1.0-F-ZnO-Group I	0.136 ± 0.0009	0.023 ± 0.0007	178
0.1-F-ZnO-Group II	0.129 ± 0.001	0.024 ± 0.0009	186
0.5-F-ZnO-Group II	0.134 ± 0.0006	0.023 ± 0.0005	180
1.0-F-ZnO-Group II	0.136 ± 0.001	0.024 ± 0.0004	178

**Table 6:** Coefficient of friction for each lubricant and achieved reduction compared to the reference lubricant.

Sample	CoF ()	CoF Reduction (%)
Reference Lubricant	0.149 ± 0.001	-
Lubricant + 0.5%OA	0.133 ± 0.001	12
Lubricant + 1%OA	0.132 ± 0.004	11
Lubricant + 1.5%OA	0.120 ± 0.001	19
0.1-ZnO	0.149 ± 0.001	0
0.5-ZnO	0.145 ± 0.003	3
1.0-ZnO	0.133 ± 0.004	11
0.1-F-ZnO-Group I	0.149 ± 0.001	0
0.5-F-ZnO-Group I	0.144 ± 0.001	3
1.0-F-ZnO-Group I	0.134 ± 0.002	10
0.1-F-ZnO-Group II	0.145 ± 0.002	3
0.5-F-ZnO-Group II	0.134 ± 0.001	10
1.0-F-ZnO-Group II	0.131 ± 0.003	12

**Table 7:** Wear scar diameter and wear scar diameter reduction for the nanolubricants and reference.

Sample	Ball Wear Scar Diameter (µm)	Reduction of Wear Scar Diameter (%)
Reference Lubricant	215 ± 3	
Lubricant + 0.5%OA	208 ± 0	3
Lubricant + 1.0%OA	206 ± 6	4
Lubricant + 1.5%OA	205 ± 8	5
0.1-ZnO	215 ± 5	0
0.5-ZnO	205 ± 3	5
1.0-ZnO	204 ± 8	5
0.1-F-ZnO-Group I	214 ± 9	0
0.5-F-ZnO-Group I	210 ± 5	2
1.0-F-ZnO-Group I	203 ± 3	6
0.1-F-ZnO-Group II	206 ± 4	4
0.5-F-ZnO-Group II	205 ± 3	5
1.0-F-ZnO-Group II	203 ± 11	6

**Table 8:** CoF and wear scar diameter for the reference lubricant and nanolubricants.

Sample	CoF ()	CoF Reduction (%)	Ball Wear Scar Diameter (μm)	Wear Scar Diameter Reduction (%)
Reference Lubricant	0.149 ± 0.001	-	215 ± 3	-
0.10-F-ZnO-Group II	0.145 ± 0.002	3	206 ± 4	4
0.50-F-ZnO-Group II	0.134 ± 0.001	10	205 ± 3	5
1.0.-F-ZnO-Group II	0.131 ± 0.003	12	203 ± 11	6
1.25-F-ZnO-Group II	0.130 ± 0.001	13	206 ± 8	4
1.50-F-ZnO-Group II	0.129 ± 0.001	13	208 ± 5	3
2.00-F-ZnO-Group II	0.127 ± 0.001	15	207 ± 4	4

**Table 9:** Elements detect in the disc surface (wt.%).

Sample	C	Cr	Fe	Si	O	P	S	Ca	Zn
Non tested disc surface	3.6	10	85.3	1.5					
Reference Lubricant	4.1		82.9		4.7	1.6		1.0	5.8
2.00-F-ZnO-Group II	5.4		58.4		25	0.9	0.5	2.3	2.9

## Data Availability

Not applicable.
